# Navigating productivity dilemmas and conflicting loyalties in activity-based flexible offices - A qualitative study of managers’ perspectives and coping strategies

**DOI:** 10.1371/journal.pone.0335945

**Published:** 2025-11-21

**Authors:** Maral Babapour Chafi, Maria Nordin, Viktoria Wahlström, Anita Pettersson-Strömbäck

**Affiliations:** 1 Region Västra Götaland, Institute of Stress Medicine, Gothenburg, Sweden; 2 Division Design & Human Factors, Department of Industrial and Materials Science, Chalmers University of Technology, Gothenburg, Sweden; 3 Department of Psychology, Umeå University, Umeå, Sweden; 4 Department of Epidemiology and Global Health, Umeå University, Umeå, Sweden; Paris School of Business, FRANCE

## Abstract

Activity-based Flexible Offices (AFOs) provide employees with a variety of workspaces to choose from based on their tasks, rather than having assigned desks. While the adoption of AFOs is increasing due to flexibility and cost-efficiency, there is limited research about the consequences of transitioning to AFOs from the perspective of staff managers. The purpose of this study is to explore how managers experience and cope with challenges that may arise in AFOs. Our qualitative descriptive study is based on two case studies that investigate the consequences of AFOs. Data collection involved semi-structured interviews with a total of 33 managers in two organisations, 12–18 months post-relocation. An inductive, bottom-up process was used for coding and thematization of the interview transcripts. Our results show that AFOs can enhance communication and collaboration depending on the units’ collaboration needs and prior geographical distribution. However, this effect was overshadowed by task-environment misalignments on within-team communication, distractions, and limitations on adjustments and recruitments. Additionally, managers faced conflicting loyalties between defending the organisation’s decision to implement AFOs while ensuring compliance with legal work environment requirements despite limited resources. There is a risk that the implementation of flexible offices will fragment and complicate managers’ tasks, such as ensuring that daily operations run smoothly, meeting legal responsibilities, and managing and recruiting staff. This poses a risk to managers’ productivity and health, and consequently, the achievement of organisational goals. The study uncovers managerial experiences, challenges, and coping strategies in AFOs, offering valuable insights for organisations considering this office type.

## 1. Introduction

The adoption of Activity-based Flexible Offices (AFOs) is increasing within public organisations. The growing popularity of this arrangement has been driven by the rapid development of information and communication technologies, which has enabled a rise in remote work, and by its flexibility and (presumed) cost-efficiency. The term AFO refers to a contemporary workplace design approach that provides employees with a variety of workspaces to choose from, based on their work tasks and activities throughout the day [1]. Implementing AFOs often involves a shift from traditional office layouts, where fixed workstations are replaced by shared spaces (ibid.). According to biannual national statistics in Sweden [2,3], the proportion of office workers in AFOs has risen from 14 per cent in 2015 to 21 per cent in 2021 [4,5].

Most studies on AFOs focus on their effects on the work environment, perceived productivity, and health outcomes from an employee perspective [see reviews in 2,3]. However, there is limited research specifically about the consequences of transitioning to AFOs from the perspective of managers. This gap in the literature is critical, as managers play a pivotal role in navigating the work environment changes that AFOs entail.

While the expected outcomes of the shift to AFOs are increased collaboration, flexibility, and improved physical work conditions [6], the shift can also introduce challenges for managers. Numerous studies have shown that increased distance between a manager and employees potentially neutralises the positive effects of a leader’s behaviour on employee job satisfaction and performance [7]. When employees choose their workspace based on tasks and preferences rather than working from assigned workspaces, the physical distance between managers and employees can increase. The consequences of a potential increase in employee-manager distance when implementing AFOs remains unanswered [8].

In general, the importance of leadership for organisational productivity and employee health is well-documented (see, e.g. literature reviews in [9,10]). When it comes to leadership in geographically dispersed settings, most studies have focused on remote work and managers’ strategies for the use of communication technologies, providing social support and regular feedback, as well as fostering trust and a positive safety climate [10,11]. While these behaviours are similar to those needed for on-site work, the communication tools differ [11], as on-site work relies more on face-to-face interactions than remote work. Whether such leadership strategies apply to flexible offices remains unclear.

The few studies that address leadership in AFOs indicate that post-relocation, employees tend to have a less positive view of leadership qualities [12]. Furthermore, these views seem to be correlated with productivity outcomes [13]. Similarly, a cross-sectional study of office types shows that employees in flexible offices have a more negative perception of leadership behaviours than those in open-plan and individual offices [14]. To our knowledge, only two studies has explored how AFOs impact leadership. Both studies show that managers experience a loss of control in AFOs compared to cell offices [15,16]. This shortcoming poses challenges for performance evaluations, as traditional presence-based evaluations fall short in flexible offices [16]. In a study on the physical work environment in AFOs, it was concluded that managers receive limited support for occcupational health and safety management [17]. However, a knowledge gap remains about how managers perceive their ability to work and support their staff in AFOs, despite the loss of control that is indicated in earlier studies. It is therefore crucial to examine the work conditions that managers face in AFOs and understand how they cope with the change and potential challenges.

The overall purpose of this study is to explore managers’ work conditions in AFOs. Specifically, we aim to investigate how managers experience and cope with the conditions provided in AFOs.

## 2. Method

This qualitative, descriptive study is a part of two larger projects about the consequences of activity-based working in public organisations in Sweden. The projects followed two public organisations that had launched AFOs (Case 1: 2015; Case 2: 2019). This paper reports on interviews with managers from the case organisations. A COREQ checklist [18,19] is used to present the study design in Appendix 1.

### 2.1. Ethics statement

Both projects included in this paper were approved by the Regional Ethical Review Boards in Sweden (2014/226–31, 2018/768–18). An additional approval was obtained from the national Ethical Review Boards in Sweden for combining the interview data from the two projects for this manuscript (2023-02461-02). The studies complied with ethical standards outlined in the Declaration of Helsinki. After informing the participants about the study, written consent for the research was obtained. All personal information is fully anonymised to protect the privacy of the participants.

### 2.2. Study context

The two organisations that participated in this study had Activity-based Flexible Offices, both with a clean-desk policy, and unassigned work areas to encourage flexible use of spaces. The majority of spaces in both cases were open areas, intended for both individual and collaborative tasks (see Table 1 for an overview of layout differences, zoning and policies in the two cases).

**Table 1:** Characteristics of the studied activity-based flexible offices.

	CASE 1	CASE 2
**Number of floors allocated to office spaces**	**2** (similar layout on both floors)	**Building A:** 5 (similar layout on all floors) **Building B:** 13 (two different layouts and zone proportions)
**Zone types and proportions**	Zoning was not formally defined but emerged spontaneously given the office’s design and layout. In total, 697 seats were provided (including lounge areas and meeting rooms), of which 0.06% (n = 44) were cell offices.	In total, 2,574 seats were provided (including lounge areas and meeting rooms), of which 0.04% (n = 102) were cell offices. **Quiet zones (5–12% of workspaces):** small open plan rooms, shared or single office rooms, and phone booths **Semi-quiet (25–32% of workspaces):** open workstation area **Collaboration zones (56–66% of workspaces):** meeting rooms, lounges, open workstation areas, small open plan rooms
**Clean-desk policy**	All employees were expected to follow a clean-desk policy and remove their belongings when they were away from the workstations.	
**Home base**	All units had an anchor point (unassigned workplaces close to lockers). The neutral design and AFO policy were intended to encourage staff to use all floors/areas despite their allocated homebase.	

**Case 1** is based on data from a municipality with a population of 56,000 and a total of 5,500 employees. The organisation relocated to new office buildings in 2015, and approximately 219 of the administrative employees (including the economics, human resources, urban planning, and education departments) moved to an AFO (detailed description in [20]).

**Case 2** involved administrative personnel in one of the provinces responsible for public services such as healthcare, culture, and transportation. The province had a population of approximately 1.7 million, and the public service organisation had approximately 50,000 employees. The organisation had implemented AFOs in two new-built office buildings, co-locating a total of approximately 1,900 employees. The relocation took place between December 2018 and June 2019 (detailed description in [21]).

### 2.3. Participants

All managers (Case 1: approx. 50; Case 2: approx. 200) who had relocated to AFOs were invited to participate. In total, 33 managers (Case 1: 8; Case 2: 25) volunteered to participate (See sample characteristics in Table 2). Most participants were women (n = 24), which was representative of the gender distribution of public sector employees. The majority were line managers (n = 23), while the rest were supervisors/team leaders (n = 3), middle managers (n = 6), or senior managers (n = 1). The number of employees per line manager and middle manager was obtained for 24 participants and ranged between 4 and 30 (average 14 employees/manager).

**Table 2:** Sample characteristics.

Demographics	Case 1	Case 2	Sum
**Roles**			
Supervisors/team leaders	–	3	3
Line managers	7	17	24
Middle managers	1	4	5
Senior management	–	1	1
**Gender**			
Women	6	18	24
Men	2	7	9
**Age range**			
30-39	1	–	1
40-49	3	5	8
50-59	2	11	13
=>60	2	7	9
Missing	–	2	2

### 2.4. Data collection procedure

We used semi-structured in-depth interviews to collect data, as this approach allows participants to answer questions freely, ensures a consistent coverage of topics, and provides an opportunity for detailed probing and follow-up questions [22]. The interview questions explored managers experiences of working in AFOs, the challenges they faced, and the support structures available to them (Appendix 2). To avoid capturing novelty effects, the interviews were held 12–18 months post-relocation (Case 1: 2017; Case 2: 2019–2020, prior to the enforcement of COVID-19 guidelines for social distancing). While the interviews in Case 1 were individual, a mix of individual and group interviews were conducted in Case 2 (group interviews were not feasible for all participants due to scheduling issues in both cases). Of the 33 participants, 13 were interviewed individually, while the remaining 20 managers were interviewed in groups of 2–5 participants (7 group interviews were held). The interviews were scheduled at a time and location convenient for the participants, with the option for face-to-face or remote interviews. Of the 33 participants, 16 were interviewed in person and 17 were interviewed remotely (some of the group interviews were hybrid with both in-person and remote participation). All interviews were led by 1–2 trained interviewers and lasted 50–145 minutes.

### 2.5. Data analysis

All interviews were recorded and transcribed verbatim. Our thematic analysis involved an inductive, bottom-up process (as described by Miles et al. [23], p. 62) of examining the participants’ reflections to find patterns and commonalities in how they experience and practice leadership in AFOs. Microsoft Word was used for coding the data. Our analytical steps involved:

1. Two randomly selected interviews were coded by three authors separately after an initial discussion about the granularity and types of codes. Two types of codes were agreed on: (i) descriptive codes that summarised the organisational context and the emotions, behaviours, and strategies described with respect to staff management in AFOs, and (ii) evaluative reflections that captured how the participants made sense of the risks and consequences of relocating to AFOs. A code could be based on varying amounts of text, from a part of a sentence to several sentences, and aimed to summarise something meaningful in the text in relation to the above code types. Of the transcribed interviews, only text that was perceived relevant to the research question was coded.
2. The codes from the sample interviews were compared and differences discussed. Overall, the granularity and the content of the codes were similar, and minor wording differences were harmonised. The remaining interviews were divided between three of the authors for coding and writing summaries for each interview.
3. The summaries were then read and compared by all the authors. This involved condensing the large number of codes from each interview into patterns (i.e., second-cycle codes, ibid. p.79) and sub-themes that had a characteristic meaning in relation to the research question. Before detailing the narrative descriptions of our findings, the results were refined in discussions among the authors and re-reading of the coded interviews.

Our coding tree is presented in Appendix 3. The findings report common patterns found in both cases with themes, sub-themes, and codes. Selected quotes were translated from Swedish to English by a bilingual author to exemplify our findings. The quotes were then attributed to participant roles (TL: team leader, LM: Line manager, MM: Mid-level manager, SM: Senior manager), as well as an identifier describing the interviewee code (I) and the specific case study (C), [i.e. “Quote” (LM-I#-C#)].

## 3. Results

**Fig 1:**
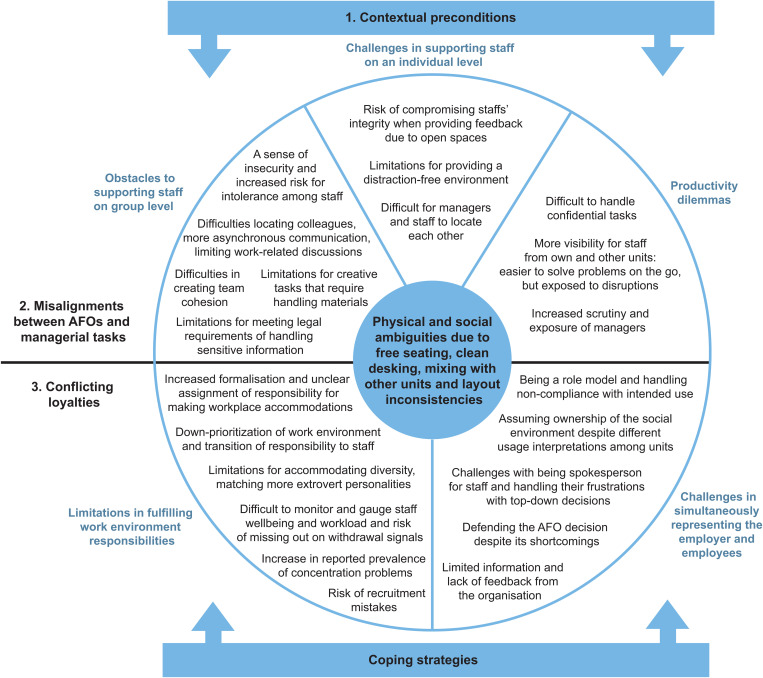
Identified themes, sub-themes, and codes that describe the managers’ narratives about their experiences and coping strategies after the move to an AFO.

The results are divided into three sections (see Fig 1). The first section summarises the identified contextual preconditions that shaped the managers’ experiences. The second and third sections present the themes that describe the managers’ experiences and the coping strategies they used to address issues that arose after the move to an AFO.

### 3.1. Contextual conditions shaping the managers’ experience of change

Three different contextual conditions were identified that describe contextual differences in managers’ work conditions: geographical distribution of staff before and after moving to the AFOs, number of employees per manager, and the office type prior to relocation.

Some units were co-located both before and after relocation. Thus, the relocation entailed dispersion of the team members, “because you don’t have a specific place where you are always together” (LM-I5-C1). Communication became asynchronous and demands increased for locating the staff. Also, coordination and planning required more time: “There were things we could solve around the table. […] Now, you have to formalize things a bit. You can’t be as spontaneous.” (LM-I32-C2). Such challenges were particularly prevalent among managers with larger groups, while those with fewer employees found it easier to coordinate and communicate with staff.

Other units were geographically dispersed prior to the move but co-located after relocation. Co-location was appreciated by the interviewees as it meant less “rushing between sites”. Communication was improved due to increased proximity, visibility, and synchronous communication: “I really like the building because you run into each other. You can exchange a few words now and then in passing, instead of accumulating things and eventually sending an email about a certain issue. […] It’s the social aspect that I think the building works very well for, being able to eliminate unnecessary friction that builds up over time (LM-I18-C2)”.

There were also partially or fully distributed units both prior to and after relocation. Managers therefore continued to rely on digital communication and planned meetings: “For those of us who have people in other locations, […] regardless of whether I had my own room before […], I use chat and write exactly where I am (LM-I22-C2)”. Despite relying on digital communication, the participants had mixed thoughts on the challenges created by the move to the AFO. On the one hand, it made it more difficult to locate one another; on the other, it enabled spontaneous interactions: “The difficult thing is that you don’t always know where people are. And it’s not just with my staff, but with everyone I need to collaborate with. At the same time, you run into them in a completely different way too. You move around; you have more spontaneous meetings, precisely because they might not be in their room (LM-I6-C1)”.

In addition, the interviewees referred to and compared the AFO with their offices prior to relocation. Those who worked in open plan offices prior to relocation found the large scale of the AFO more difficult to navigate: “With the 1700 people [here], you don’t even have a chance to get to know everyone” (TL-I23-C2). According to those who moved from cell offices, AFOs allowed for better visual control without “the closed-off feeling in a traditional corridor”, while cell offices were more flexible regarding furnishing: “It was constantly used as a meeting room. Either for team meetings or check-ins. When I wasn’t there, my employees could use it. […]. It worked very well and efficiently. But today, this [office] doesn’t work” (LM-I28-C2).

### 3.2. Misalignments between AFOs and managerial tasks

The interviewees highlighted challenges related to supporting their staff while maintaining their own administrative productivity and working on tasks that require concentration.

#### 3.2.1. Obstacles to supporting staff on group level.

The AFOs predominantly comprised open spaces shared between several units. These spaces included areas for individual or side-by-side work and lounges located in proximity to each other without clear boundaries. Such ambiguous boundaries created a sense of insecurity and discomfort among staff, as they did not know “where to sit during the day”, whether they would be working apart or side-by-side, or where it was appropriate to talk to colleagues without “disturbing others”. The interviewees described that the social agreements for different zones were “easy to forget” and introduced a risk for conflict: “We don’t treat each other kindly when it’s messy. It’s crazy that we need rules at the workplace, instead of liking each other and having patience and understanding for each other” (LM-I28-C2).

The managers pointed out that the ambiguous boundaries limited work-related conversation and knowledge exchange, which made it more difficult for team members to support one another and thinned out unit discussions. Furthermore, spontaneous interactions were replaced with asynchronous communication or planned meetings. Additionally, staff located elsewhere had limited access to AFOs, even if they were part of a unit inside the AFO (in contrast to previous offices in which all staff were welcomed). Thus, AFOs were perceived to decrease flexibility and spatial support for communication. The interviewees reported an increased workload for managing units and creating team cohesion.

**Long-serving manager: “**The demands have increased significantly. I check my emails almost around the clock. Since we see each other less, I have less visual contact. Instead, I write emails. It would have been easier to have designated areas to have dialogues and work together.” (LM-I13-C2)

**Recently appointed manager:** “Challenging from a personnel responsibility perspective; not having the unit gathered to get to know each other. […] It would have been easier if we sat close, simply to see each other naturally.” (LM-I8-C1)

Supporting the units in their assignments was another challenge for the managers in the AFOs. Ambiguous boundaries and inconsistencies in zone functions were reported to limit creative discussions for problem solving among staff. Interviewees also mentioned that AFOs did not provide adequate conditions to meet confidentiality and data protection requirements, in units whose main responsibilities dealt with sensitive information, for example, patient data, procurements, or building permits. Additionally, the lack of assigned workstations and clean-desk policy did not support tasks such as infrastructure planning drawings and the production of creative content for communication purposes. The interviewees expressed a need to be able to use workspaces for a longer time period, so that they did not need to install and remove project material on a daily basis.

**Manager of a unit working with technical drawings:** “The office is not designed for collaboration, for discussions around drawings, screens, and whiteboards, which is the main task. Meeting areas with curtains disturb the entire office, and there are no spaces for small group collaborations where you don’t disturb others.” (MM-I3-C1)

***To cope with obstacles to supporting staff on the group level***, the managers adopted two types of strategies. **First**, they found other locations and negotiated unit-specific spaces in the AFOs. This changed the environmental conditions for the managers’ units. This strategy was mostly used by those with legitimate power (i.e., higher positions) or those with expert power (working with technical or legal expertise), who had more power to negotiate: “When we were trying to get some quiet rooms […], I passed by the facility management group and took the opportunity to ‘speak out’ about it not working so well for us. […] We manage the organisation’s financial capital […]. One might think that would be quite important” (LM-I28-C2). Those with a limited ability to negotiate resorted to adopting non-compliant behaviours, such as claiming spaces, which challenged the AFO concept. The **second** type of coping strategy was creating “control systems”, such as increasing asynchronous communication, clearly communicating availability, communicating whereabouts to make it easier to locate one another, formalising social activities, bringing together the staff via tasks instead of location, and holding more frequent staff meetings:”I have meetings with them every week” (LM-I20-C2).

**Manager of a unit working with procurement expertise: “**We are the only unit in the building that has been given its own room, […] because we have confidential material, product samples, and such. […] My immediate boss says: ‘yes, you have my full support.‘” (LM-I2-C1)

#### 3.2.2. Challenges in supporting staff on an individual level.

The managers noted that they were unable to provide a distraction-free environment for staff who struggled with tasks that require concentration. The openness of the workspaces and lack of boundaries between different work zones and social areas (as well as mixing with other units) created distractions: “You can’t put up a sign saying: work in progress!” (LM-I28-C2). The free seating arrangement made it difficult to locate each other: “My secretary often sits somewhere completely quiet to write meeting notes and such. I can’t just say: Let’s go for coffee! It’s a bit more complicated. I don’t know where she is” (MM-I25-C2). The managers described this as a problem when employees chose to isolate themselves due to poor preformance or mental ill-health. Yet another problem with the open workspaces was phone calls from stakeholders such as customers and/or the public. Staff were not accustomed to taking phone calls with colleagues nearby, but they needed to remain in the open workspace to have access to information on their computers during the phone conversations: “It was very stressful in the beginning. […] They felt they were disturbing others when taking phone calls among others because they need the computer screen to be able to see where the customer is” (LM-I24-C2).

AFOs were perceived to provide limited support for managers in their communication with staff. Being under scrutiny in open spaces where they were mixed with other units created a number of challenges, particularly for one-on-one communication with staff. The interviewees described that providing feedback to staff was more complicated in the AFO and imposed a risk that an individuals’ dignity would be compromised: “Everyone knows what you are doing. And before I understood that, maybe someone felt a bit singled out” (LM-I24-C2). Interviewees reported that cell offices were more conducive to spontaneous interactions, as managers could drop in without drawing attention to a single individual. The limited number of enclosed individual and meeting rooms was another obstacle to providing spontaneous feedback.

“If you want to give someone [prompt] feedback, positive or negative, it becomes so incredibly serious, you have to single them out and find a nook […]. So, often you have to say that it’s nothing serious. […] It creates curiosity among others.” (LM-I6-C1)

***To cope with difficulties supporting staff on individual level,*** the managers devised different strategies. **First,** they used a combination of behavioural and environmental strategies. Examples of behavioural strategies included being more observant to stress signals, determining when staff were available and could be interrupted, sacrificing their own needs for quiet spaces to leave space for staff, and accepting that distractions were inevitable in open spaces when communicating with staff. Environmental strategies included rearranging the furniture to provide privacy, helping staff locate quiet areas (Case 2 where formal zones were available), and allowing work-from-home. **Second**, a combination of passive and active strategies was used to cope with the loss of visual control. Apart from acceptance, managers described focusing on “follow-up on performance” or actively “touring around” the facilities, intentionally choosing central locations to “signal availability”, or rotating workstations to meet the staff and prevent unintentional favouritism: “I don’t sit at the same workplace every day, [...] just so that it’s not the same employee sitting next to me” (SM-I15-C2). **Third**, the managers described using communication strategies to avoid compromising dignity and privacy, for example, planning frequent individual meetings.

**Manager coping with environmental stressors: “**I have to try to see when [my staff] feel stressed or think it’s too much and also consider what in the environment makes them feel stressed […]: ’Is it that you might need to find a room or work from home?’ It feels kind of politically incorrect and maybe not so great from a work environment perspective to send someone home, but there aren’t that many alternatives.” (LM-I6-C1)

**Manager coping with risks of compromising staff dignity:** “You have to be careful, even if you are in a [private room]. You can’t feel certain that you are alone, because it’s so poorly soundproofed that people outside have no problem listening in on a discussion. You have to get used to talking very quietly.” (LM-I2-C1)

#### 3.2.3. Productivity dilemmas.

Interviewees described that AFOs required them to be more available. This increased availability introduced a productivity dilemma that required balancing visibility and accessibility with the need for undisturbed work. Exposure and proximity to others in open workspaces meant navigating increased scrutiny and compromised tasks that required handling sensitive information such as personnel data: “You sit so close and the screens are so big that I don’t always feel like I can do everything I need to […, with] my employees sitting there, because I’m working on a rehabilitation case that gets exposed” (LM-I32-C2). In combination with the disruptions caused by open workspaces and time spent finding a workstation, this led to more cognitive effort. Managers had less time to focus on actual tasks, which increased their stress levels while decreasing productivity: “I get disturbed almost everywhere—that’s how it is as a manager” (LM-I32-C2). Managers reported that they were always under scrutiny and perceived that there was a risk of signalling a weak leadership. There was thus a risk that they would be the targets of workplace gossip if they stood out or did not adhere to social agreements: “Weak leadership is more apparent now. If you don’t dare telling your employees that: ‘this isn’t working, we’ve agreed on something else, you actually have to do it this way’” (LM-I5-C1). However, one positive effect of being exposed to others was the feeling of being more productive in terms of staff management responsibilities and tasks, as managers were able to find people and solve problems on the go.

“Previously, I could go and settle in at my place between meetings. That settling between meetings is very difficult now, and it takes a lot of transition time. A difficult dilemma is: should I be on the [home base] floor? Because then I know [staff] can find me, and I can see them and get a sense of how things are going. Or should I sit somewhere else where I can be a bit undisturbed?” (LM-I31-C2)

***To cope with the risk of productivity loss***, managers adopted four types of strategies: compliant behaviour, non-compliant behaviour changes, boundary setting strategies, and boundary expanding behaviours. Compliant behaviours are exemplified by “training oneself” to ignore distractions, such as people passing by, by “using headphones” to signal a need for concentration, and by booking meeting rooms (even when they had no meetings) to ensure they would have access to private spaces for uninterrupted discussions. Some managers mentioned non-compliant behaviours, such as claiming single rooms, but added that this was easier for those with tenure or higher positions, who have more support from senior leadership. Boundary setting behaviours were exemplified by the use of quiet zones for focused work and using clear boundaries: “I think the quiet zone is quite wonderful. […] It’s a signal to others that: I want peace and quiet now, don’t disturb me with anything!” (TL-I23-C2). However, quiet zones were not appreciated by all managers: “Every time the door opens, you naturally get disturbed. Most of us at least take a peek to see if it is someone you’ve never met: Who is this? Do I know him or her? Should I ask how they are?” (LM-I28-C2). Thus, balancing visibility and accessibility with the need for undisturbed work remained a dilemma. Another strategy was therefore to expand the work boundaries, that is, work-from-home or working longer hours to find quiet time for focused work.

“I want to be available for the staff, so I don’t go and sit in a quiet zone. […] You can’t sit in there as a manager, and that also makes administrative tasks difficult to perform during normal working hours. And then it’s very easy to postpone it; either take it home or make sure to be here early so you can do it before others arrive.” (LM-I20-C2)

### 3.3. Conflicting loyalties

The theme conflicting loyalties describes the dilemmas that the managers described in their roles. The sub-themes were limitations in fullfilling work environment responsibilities and challenges in simultaneously representing the employer and employees.

#### 3.3.1. Limitations in fulfilling work environment responsibilities.

The managers faced challenges in fullfilling the responsibilities imposed on them by the Swedish Work Environment Act (e.g. monitoring and gauging staff wellbeing and providing support). This was a consequence of the fact that the AFOs provide an opportunity for staff to isolate themselves and remain hidden by choosing enclosed workstations or floors other than those designated for the units. The ability to choose workspaces away from the unit posed a risk that individuals would be excluded from informal activities and feel isolated from the group. As a result, the managers highlighted difficulties in finding a balance between trust and control: “We have low demands about informing [us] about where you are sitting […]. If I have an employee who I can’t really keep track of and who doesn’t like to sit on [our floor…], it becomes very difficult to say: ‘I want you to sit here.’”(LM-I32-C2). Thus, managers felt they had to actively seek out employees and gauge their wellbeing.

“Those who don’t feel comfortable withdraw and disappear. And then I, as a manager, have to seek them out, look for them, and book a time to find that employee. I don’t naturally see what’s going on with that employee either because it’s […] very easy for that person to, let’s say, put on a happy face. […] It’s very difficult as a manager to detect, because it’s harder to catch things that aren’t working.” (LM-I28-C2)

In terms of taking on responsibility for the physical work environment, the interviewees felt conflicted between addressing individual needs for adjustments and the constraints that AFOs entailed. The interviewees described that they, as well as their staff, found it difficult and “time-consuming to adjust workstations” or move around their equipment. Ergonomic aspects were described to be underprioritised and subject to compromises, in contrast to having occupational health and safety experts adjusting assigned workstations. Thus, the responsibility for the physical work environment was transferred from the employer to employees: “It becomes an individual responsibility to think about adjusting the screen light, knowing where the buttons are, somehow considering – is this a good chair for me or not.” (LM-I32-C2)

According to the managers, concentration problems, noise sensitivity, migraines, etc. had increased since moving to AFOs. They found it more difficult to handle such isues in AFOs due to the open spaces and the limited number of private rooms. The managers had to balance between the legitimacy of employee requests and allocating the few accessible private rooms as a workplace adjustment: “Everyone could probably say: but I can’t concentrate, I have difficulty concentrating. […] It sounds harsh, but sometimes it needs to be black and white (LM-I1-C1)”. Also, once an adjustment was made, its legitimacy had to be reassessed regularly: “What’s difficult is where we draw the line in the end. How far, when should we start, and when should we stop adapting? What’s okay and what’s not?” (LM-I21-C2). Another sensitive topic was protecting an employee’s dignity when assigning workstations without undermining other units to access these spaces.

The managers stated that their traditional offices were superior to the AFOs when it came to making workplace adjustments. Instead of addressing the physical work environment problems in a prompt and flexible manner, the AFOs required formalised processes with the involvement of different stakeholders, which led to an increased administrative burden. In addition, the boundaries of responsibility for the physical work environment in AFOs were perceived as vague. Due to the fact that spaces were now shared across units, the responsibility and mandate shifted from the individual managers to a collective of managers: “I was much more clearly responsible for that desk than for all the desks” (LM-I5-C1). These limitations created a sense that statutory work environment responsibilities were not being adequately addressed, which in turn triggered stress.

“We controlled our own operations in the old offices. We could very quickly take actions. […] Now, I have to involve an ergonomist who creates a report that then goes to those who make decisions in the building. Then you might get the okay to place a wrist support ‘on that spot for this amount of time, before we do an evaluation’. Just finding that path creates stress for me as a manager.” (J-I5g-C2)

Challenges were also raised regarding how AFOs undermined the inclusion of certain personalities, favouring extroverts over introverts. Those who are unable to handle distractions have to compete for the limited number of private rooms. The managers described that social agreements for sharing workspaces were disregarded by “more assertive individuals”, who claimed more space, while others “hold back, as they don’t want to cause a stir; they don’t want to be bothersome” (LM-I5-C1). According to the managers, both staff and managers were required to be ”very social, with a desire to be visible and on-stage”. Limitations for accommodating diversity led some managers to resign:”I am experiencing more stress; even more than the high levels I had before. After 30 years in this role […], I have actually resigned. And I am not the only manager who is leaving (LM- C2)”. Along the same theme, additional demands involved recruiting new staff with personalities suited to coping with the environment:

“This was a question in the interview when we were hiring: How do you feel about working in such an environment? We had a candidate who was very qualified but said that this was problematic; she was easily disturbed, and it was important for her to have peace and quiet. She didn’t get the job. Instead, we chose a person who said that they felt comfortable with this. […] It scares me when I think that I may have made a hiring mistake.” (MM-I33-C2)

**To cope with the challenges of fullfilling statutory work environment responsibilities in AFOs**, the managers reported using different strategies. By “being more observant”, they learned about staff preferences for work locations, increasing their ability to identify those who isolated themselves. They also booked regular one-on-one meetings so that they could assess individuals’ wellbeing: “I want to see my staff face-to-face, because you can see how they are doing better than if you contact them [remotely…]” (SM-I15-C2). Adressing ergonomic issues was another coping strategy used to encourage behaviour change among staff. Managers also sought help from occupational health and safety experts to handle these challenges: “I have received help from our physiotherapist, who was here to look at how staff work and what they need […] and show how to adjust chairs” (LM-I24-C2). Some mentioned more passive and resigned coping strategies, such as reduced engagement: “You almost have to let go of the idea of keeping track of employees. It is not possible. […] According to the Work Environment Act, you have to take responsibility for your employees’ wellbeing as a manager. How can you do that if you don’t even see them?” (TL-I23-C2). Managers also strived to ensure staff would be undisturbed while performing their taks by focusing on individual behaviours, for example, “touring with staff to help find quieter spaces”, promoting “social agreements so that quiet zones remained quiet”, allowing remote work, and formalising processes for workplace adjustments.

“We have tried to gather together in a forum […] to capture all questions, channel them, and try to address them as much as possible. And sort out what we can do and what facility [management] should take care of.” (LM-I31-C2)

#### 3.3.2. Challenges in simultaneously representing the employer and employees.

Working in a dual role was another challenge that managers discussed. This involved representing the organisation and being a spokesperson for staff before, during and after relocation to the AFOs. For example, they had to communicate and defend the organisational change despite the shortcomings of AFOs and limited information about ongoing building changes: “It’s difficult, or rather impossible, to communicate it to employees when you don’t know about it yourself. That’s a flaw in this work environment” (MM-I25-C2). Communicating and defending the AFO and its limitations required “courage to put one’s foot down and say this is the work environment that we have chosen. […] You have to wear the employers’ hat” (LM-I8-C1), implying that managers need to be loyal.

To meet their work environment reponsibilities, managers had to handle irritation and frustration among staff and negotiate with facility management. Negotiating with facility managers for adjustments and suggesting changes was described as an unpleasant process: “I felt I was very bothersome and whiny and felt very uncomfortable (LM-I28-C2)”. It also required taking other units into consideration: “Other departments heard about this [referring to an assigned quiet area for the unit], and then there was friction. They felt they were at a disadvantage” (LM-I28-C2). Managers described that, at times, their requests were disregarded and could be seen as ”pointless”, as decisions were made on a higher organisational level without consideration for employee needs: “It’s really difficult to be that link between employees and some sort of official-good- employer-representative. I know there will be no response if I tell my employees to send a proposal to facility management.” (LM-I5-C1)

Adjusting to the organisational change meant assuming ownership of the social environment. Managers described that they had to establish new ways of working, serving as an encouraging role model and, at times, confronting staff to ensure that they adhered to expected ways of working: “We, leaders, have some responsibility to create the atmosphere that we want […]. You should practice what you preach [… and] make sure that employees do what we have agreed upon. That is my responsibility” (LM-I5-C1). Several difficulties were raised regarding social agreements and expected ways of working. The large number of ambiguous “rules and routines” left room for interpretation, and organisational support for the needed behaviour changes was limited to documents and signage: “New habits don’t just happen because you put a note on a table” (LM-I14-C2). Despite initial ambitions of synchronizing behaviour to make all floors accessible to all employees, different units had different social agreements and norms for using spaces, which made it difficult to handle non-compliance.

“I don’t know who is responsible for what in the building. It’s still very unclear to me. I don’t know who to ask about different things. I don’t even know where to read about different things. […] What are the rules? What can you do? What can’t you do? Sometimes I think people talk too loudly, and then you can say: ‘try to keep it down a bit’. If they don’t stop, who should I talk to? Who is their manager? I don’t know how to handle that.” (MM-I33-C2)

**To cope with the challenges of representing both the employer and the employees in the AFOs,** managers used different strategies. The managers strived to maintain a “positive attitude” and “switch workstations frequently” to be a role model for the organisational change. They also tried to help staff see the positive aspects of AFOs, instead of fixating on the negative aspects. Some managers mentioned sacrificing staff or unit needs for adjustments to ensure fairness across the units. They also pointed to the need for formal and informal forums to discuss work environment problems and solutions. Such forums were created on both local and central levels in Case Two: “…with union representatives and our HR and all unit managers, who met with some regularity, […] where we talked about the work environment challenges and the change work it entailed” (LM-I31-C2). Participation in these forums allowed for closer collaboration with facility management which increased negotiating capacity. Managers in Case One also voiced a need for such forums in order to raise work environment issues to an organisational level.

## 4. Discussion

The overall purpose of our study was to explore how managers experience and cope with the challenges of relocating to activity-based flexible offices. Our results show that AFOs can enhance communication and collaboration depending on the units’ collaboration needs and prior geographical distribution. However, this was overshadowed by task-environment misalignments posed by AFOs in terms of within-team communication, environmental stressors such as noise, and limitations for adjustments and recruitments. In addition, managers faced conflicting loyalties between defending the organisational decision to implement AFOs and ensuring unit productivity and compliance with legal work environment requirements with limited resources. Our discussions will address the identified productivity dilemmas and conflicting loyalties that managers faced.

### 4.1. Productivity dilemmas in activity-based flexible offices

Our study shows that privacy is perceived to be crucial for productivity, and a lack of privacy can overshadow the intended communication and collaboration gains in AFOs. Our findings illustrate the broader organisational challenge of balancing short-term efficiency with long-term adaptability, known as the “productivity dilemma” (cf. Abernathy, 1978 as cited in Adler et al. [24]). Here, we found that optimising office layouts for efficiency with respect to single tasks can make the environment rigid and less adaptable to the interdependencies and transitions between different tasks of both managers and their staff. The managers’ views in our study are also in line with previous research on AFOs. For example, a recent study found that employees rated their workplace environment less favourably in terms of teamwork, concentration, and productivity when they had a higher number of desks within their field of vision and when they faced away from the room with a large area behind their back [25]. Another study found that shared workspaces lead to more distractions, less privacy, and, contrary to intentions and expectations, lower collaboration levels than cell offices [26]. Rather than increasing collaboration, distractions and a lack of privacy had a negative effect on performance [ibid.]. Similarly, lower cognitive ratings are reported in open offices due to higher noise levels [27]. Another study of AFOs showed a 20 per cent decrease in cognitive performance in collaboration zones compared to quiet zones [28]. These findings underscore the importance of providing a balance between spaces for single-task efficiency, such as quiet areas to enhance concentration and productivity, and exploring spatial solutions that cater to the overall roles of employees and the interdependencies between their tasks.

The managers’ narratives demonstrate that relocation to AFOs increased proximity to other units. On the other hand, the managers also reported an increased distance with respect to their own staff. This duality may also present benefits and challenges when on-boarding new managers, as the environment may help managers gain a broader understanding of the organisation as a whole, while also posing challenges in establishing team cohesion and interacting with staff. Social relations are vital for well-being and health [19,29]. The theory on social relations and social support has primarily focused on individual and group factors and less on the importance of the physical environment. This was remedied in a study on social affordances in AFOs [30], showing that lack of space, and resulting feelings of crowding and exposure to noise, had a negative impact on social interactions. Another study showed that collaboration tends to decrease exponentially with increased physical distance in open plan environments [31]. Additionally, it has been shown that it is more difficult to form new social ties in open-plan offices, and already established work-related social ties tend to decrease in such office types [32]. Thus, the intended social benefits of AFOs may be undermined by physical factors.

Managers also feared making recruitment errors, as they tended to focus on whether prospective employees could adapt to an AFO. Flexible offices can therefore inadvertently foster groupthink if individuals who can adapt to the environment are favoured over highly skilled individuals who prefer more traditional work settings. According to one study, individual factors influencing satisfaction in AFOs are: need for belonging, need for privacy, work autonomy, social interaction, internal mobility, and age [25]. We hypothesize that brain drain can occur when the work environment does not support diverse working styles and needs, resulting in loss of institutional knowledge and expertise and productivity. To mitigate these risks, organisations should prioritise creating flexible work environments that accommodate various working styles, psychological needs, and preferences.

Our findings show that managers increasingly feel that they are “on-stage” and subject to scrutiny due to the fact that they share the premises with other units. Concerns that managers would signal weak leadership were also raised in relation to establishing new ways of working, as employees who do not follow the agreed rules can continue to do so, which affects the overall success of AFO implementation. At the same time, managers were to create conditions that would support productivity in their unit, which at times required disregarding social agreements. One study suggests that strong change-oriented leadership can mitigate productivity declines during transitions to AFOs [13]. It is therefore crucial to provide managers with organisational support so that they can effectively implement change management and navigate productivity dilemmas in AFOs.

Our results show that managers adopted various individual and organisational coping strategies to mitigate productivity losses in response to a sub-optimal work environment. Individual strategies included techniques to help manage environmental stressors and change negative thought patterns. Organisational strategies included open communication, recognition of issues, training, and support networks. The identified strategies represent both problem-based and emotionally focused strategies [26]. The managers showed well-developed executive abilities in applying problem-based strategies to solve problems that arose in the AFOs. Our findings also suggest that the effectiveness of coping strategies varied across managerial tiers. Managers with legitimate power (typically those in higher-ranking positions or those with expert power) were more successful in negotiating unit-specific spaces and influencing environmental adaptations, e.g. securing quiet zones or dedicated meeting areas for their teams, citing operational necessity. As a result, not all issues could be resolved for the line-managers, either because the physical environment did not support the solution, or in absence of legitimate power, or due to the absence of organisational support. This led to feelings of frustration and other negative emotions, which managers had to deal with themselves. The need to apply coping strategies taxes an individual’s resources, regardless of whether these are problem-based or emotionally focused [27]. Therefore, to enable managers to achieve balance, employers should review working conditions with attention to different managerial levels. This review should ensure that the energy used to cope with the challenges of an AFO does not drain managers of the energy they need to balance their work duties. Interventions may involve leadership development programs or forums for lower-tier managers, to provide avenues for support, feedback, training and access to shared resources that could help mitigate the uneven burden of AFOs across the managerial hierarchies [33–35].

Our findings indicate risks posed to managers’ productivity and health, and consequently, the achievement of organisational goals. Having the same level of responsibility with inadequate physical resources creates an imbalance between job demands and resources [see JD-R in 36]. A sustained imbalance between high job demands and low resources leads to increased effort depletes employees’ physical, emotional, and cognitive resources and may lead to exhaustion, burnout, turnover, and poor service quality, particularly in human services [37]. The rise of flexible and hybrid work arrangements necessitates a re-examination of JD-R model’s applicability in contemporary work environments [38]. New ways of organising work introduces novel demands on both employees and managers, including increased cognitive load, fragmented communication, and the need for self-regulation and boundary-setting. Organizational support emerges as a critical resource in hybrid contexts. Without sufficient investment in digital tools, training, and inclusive leadership practices, the potential benefits of hybrid work may be undermined.

It is relevant to emphasize that our participating organisations operate within the public service sector, where evidence points to hazardous psychosocial working conditions and high levels of stress and sick leave [39,40]. Managers, in turn, constitute part of the subordinates’ work environment and impact employee health and turnover rates [41,42]. Research has also shown that managers experiencing poor working conditions are more likely to make less informed decisions and provide insufficient support for their subordinates, which can negatively influence the organization’s productivity [43,44]. Therefore, when modifying physical work environment, we recommend prioritizing improvements to managers’ working conditions, as a key strategy for promoting both employee well-being and organizational performance.

### 4.2. Conflicting loyalties

In the narratives, an experience of ethical stress is expressed. Ethical or moral stress can be defined as a psychological state arising from a person’s uncertainty about fulfilling relevant moral obligations [45] and is a frequently studied phenomenon among healthcare professionals [46]. Among managers, this uncertainty often emerges when managers face competing demands for limited resources from multiple stakeholders or across various role identities [47]. Similarly, our study shows that managers in AFOs deal with competing demands and limited resources (e.g. distraction-free spaces), simultaneously balancing the needs of their own staff with staff in other units and defending the organisation’s decision to implement AFOs without clear guidance. Our results are in line with two other case studies of AFOs, where managers reported a lack of support for making workplace adjustments [17,48]. Thus, fulfilling the legal requirements for the management of occupational health and safety in Sweden is more complicated for managers in AFOs, resulting in ethical stress.

On an organisational level, a broad range of challenges are at play in terms of workplace design. In particular, societal and environmental challenges (e.g. the United Nations’ 2030 Agenda with its sustainable development goals) add another layer of complexity. From an environmental perspective, these goals require the efficient use of energy and workspaces, particularly when staff work from multiple locations both within and outside of an organisations’ facilities. However, our results indicate that transitioning to AFOs imposes risks related to productivity loss and work-related health problems, presenting a conflict between environmental and social sustainability goals. Organisations must recognise and manage these “wicked problems”, which involves navigating complex trade-offs, fostering collaboration, and understanding that progress in one area may impact others. Despite the rhetoric of staff empowerment when implementing AFOs, the dominant organisational agenda for implementation has been cost management. Parker [6] stressed the importance of highlighting and further examining several aspects of these competing goals.

Creating a satisfactory physical work environment when it comes to lighting, furnishings, acoustics, and layout improves employees’ perception of organisational support. This allows employees to participate in both job crafting and decision making, and in turn increases workers’ engagement and organisational citizenship behaviour [49]. Thus, investing in well-thought-out, employee-centred office design is crucial when planning new offices, as it is less expensive to make changes on blueprints than to alter physical structures at a later date.

### 4.3. Study relevance and practical implications in the post-pandemic era

Conducted before the COVID-19 pandemic, our study show that remote work was used to cope with the shortcomings of AFOs. Regardless of the degree to which remote work was practiced, managers in AFOs struggled due to a limited overview of the office, increased asynchronous communication, and the need for more scheduled meetings. These challenges align with findings from studies on remote work. For instance, one study shows a decrease in synchronous communication among remote workers [50]. A recently published study [51] describes how remote work in the post-pandemic era requires changes in communication patterns for collaboration, employee engagement, and the sense of belonging. The authors also emphasise the importance of leadership in creating a successful remote work culture in remote or hybrid work. In line with this, we found that managers who had partially or fully distributed units both prior to and after relocation had developed leadership strategies to rely more on digital communication and to strive for a balanced approach between trust and control. Despite these strategies, they reported that the AFO setting made it more difficult to locate staff and other colleagues when at the office. Working as a manager in an AFO environment that is combined with hybrid work could therefore pose even more challenges to the development of successful leadership strategies. We argue that organisations should be aware of and find ways to support managers to develop their leadership strategies when combining flexible offices with hybrid work. For example, organisations can offer leadership training that focuses on managing distributed teams, fostering team cohesion across physical and digital spaces, and navigating the challenges of reduced face-to-face interaction in flexible offices. Other examples may include support from supervisors for prioritization of work by means of a dialogue about what should and should not be done when physical and technical resources are inadequate. Peer forums or mentoring programs can also provide managers with opportunities to share experiences and learn adaptive strategies from colleagues in similar roles. Additionally, organisations can implement clear communication protocols and digital collaboration tools that help managers maintain visibility and engagement with their teams, regardless of location. Apart from these recommendations, the physical environment should provide access to quiet zones or bookable private spaces within flexible offices for supporting managers’ tasks. By combining environmental adjustments with strategic leadership support, organisations can help managers lead effectively in increasingly flexible and complex work settings.

Reflecting on our findings in the post-pandemic era, the increased opportunity for remote work could compensate for an office environment that does not meet employees’ needs for privacy and concentration. In such cases, remote work could protect against the lack of supportive office environments or unwanted social interactions. On the other hand, all staff may not have the spatial or social conditions for remote work, which means that employers still need to offer supportive office environments. The increased autonomy associated with remote work has been shown to lower the risk of burnout [52], and remote work could be a suitable strategy for employees who struggle to be productive in the office. Another recent study found that staff who worked more remotely than they would like reported worse well-being compared to those who worked less remotely than they preferred [53]. These studies suggest that remote work should be carefully managed [52,53]. If remote work increases, this will likely also influence managers’ strategies for fostering strong communication and collaboration in the workplace. This shift could also complicate the sense of belonging while exacerbating inequalities among people with disabilities [51,52]. We believe that it is essential that the work environment provides the necessary conditions, so that remote work does not have to be used to manage one’s tasks.

### 4.4. Methodological considerations

One of the strengths of our study is the choice of interview method, which allowed us to capture managers’ experiences in AFOs, adressing a knowledge gap in workplace studies. Another strength is our sample of two organisations, with a total of 33 managers from different work contexts.

To assess the rigor of our study, four methodological criteria relevant for obtaining trustworthiness in qualitative research will be discussed (see Miles et al. [23], p. 304). **First**, credibility and neutrality were ensured by using multiple interviewers in the two case studies and by performing several re-readings of the transcriptions. This allowed for careful labelling and comparison of codes and themes. **Second**, confirmability was achieved through frequent discussions among all co-authors and through presentations and discussions of results within the research group. **Third**, dependability was strenghtened by recruting participants 12–18 months post-relocation. This ensured that we would capture long-lasting aspects of managers’ work conditions in AFOs and avoid capturing initial impressions of working in AFOs. Despite mixing individual and group interviews, we found similar narratives and a saturation of data from both case organisations. Individual interviews tend to generate a broader range of unique responses per person [54], likely with more sensitive information than group interviews [55,56]. As the purpose of our study was exploratory, we found that mixing these methods allowed us to uncover different types of experiences and capture both moderately and very sensitive topics. Another consideration in terms of dependability is self-selection bias, as our participants volunteered to share their insights and experiences, and may have overrepresented certain viewpoints. As our participants from both cases represented a variety of departments, we found that their in-depth and detailed narratives reflected how it is to lead different types of work groups in AFOs which corresponds to our exploratory aim. A purposive sampling would have been devised if we did not have representation from different parts of the organisation, or if our focus was on a certain managerial level. It is important to note that we have an overrepresentation of line-managers in our sample (portraying the organisational structure), and therefore experiences of mid- and top-level management are underrepresented in our study.

To achieve transferability, efforts were made to obtain a rich variation in the study sample with respect to the recruitment of managers with different characteristics. This included prior experience as a manager, number and distribution of employees, type of work unit, and office type prior to relocation. In addition, we recruited interviewees from two public sector organisations with different assignments, AFO layouts, and geographical locations. The timing of our data collection prior to COVID-19 presents a risk for limited transferrability of our findings to AFO contexts in which remote work is common today. However, it is important to note that remote work was raised as a strategy by some managers for coping with distractions. In additon, some of our interviewees were responsible for geographically distributed units and thus managed remote workers. The physical distribution of staff in the AFOs and the resulting challenges identified in our study are similar to the challenges of increased distance between remote workers and their managers, which is why we believe that our findings are also transferable to managers in AFOs in the hybrid era.

## 5. Conclusions

The overall purpose of our study was to explore how managers experience and cope with the challenges of relocating to activity-based flexible offices. There is a risk that flexible office environments will fragment and complicate managers’ tasks in terms of ensuring that daily operations run smoothly, meeting their work environment responsibilities, and managing and recruitment of staff. This poses a risk to managers’ productivity and health, and consequently, the achievement of organisational goals. What is lost in spatial support provided by the physical environment needs to be compensated by organisational support, through strategic leadership support and training.

## Supporting information

Appendix 1 COREQ checklist. (DOCX)

Appendix 2 Questions used for the semi-structured individual and group interviews with the managers. (DOCX)

Appendix 3 Coding tree with main themes (numbered), sub-themes and the codes. (DOCX)
