# Supplementary material for: Navigating productivity dilemmas and conflicting loyalties in activity-based flexible offices - A qualitative study of managers’ perspectives and coping strategies
Source: PLoS One. 2025 Nov 21;20(11):e0335945. doi: 10.1371/journal.pone.0335945 (PMC12637956; doi:10.1371/journal.pone.0335945)
Supplement: Appendix 1 — (DOCX) [file pone.0335945.s001.docx]

**Supporting information: Appendix 1. COREQ checklist**

| **N** | **ITEM** | **QUESTIONS** | **DESCRIPTION** |
| --- | --- | --- | --- |
| **Domain 1: Research team and reflexivity** | | | |
| ***Personal Characteristics*** | | | |
| **1** | Interviewer/ facilitator | Which author/s conducted the interview or focus group? | Interviews in Case 1 were held by APS and MN. Interviews in Case 2 were held by MBC and a consultant. |
| **2** | Credentials | What were the researcher's credentials? *E.g. PhD, MD* | All authors have a PhD degree. |
| **3** | Occupation | What was their occupation at the time of the study? | All authors were full-time researchers. |
| **4** | Gender | Was the researcher male or female? | All researchers were female. |
| **5** | Experience and training | What experience or training did the researcher have? | Training regimes for all the interviewing researchers included plentiful instructions in qualitative data collection and analysis. They have previously carried out interview-based studies about physical work environment, design, organisational ergonomics, psychosocial work environment, and occupational health. |
| ***Relationship with participants*** | | | |
| **6** | Relationship established | Was a relationship established prior to study commencement? | A relationship was established with a contact person at each participating case. The contact person then facilitated the recruitment of interviewees by providing the researchers with contact information for individuals in the sample. |
| **7** | Participant knowledge of the interviewer | What did the participants know about the researcher? E*.g. personal goals, reasons for doing the research* | All participants received information about the project. They were introduced at the beginning of the interview to the goals and procedure of the research project, both in writing and from an intro script read at the beginning of each interview. |
| **8** | Interviewer characteristics | What characteristics were reported about the interviewer/facilitator? E.g. *Bias, assumptions, reasons and interests in the research topic* | A personal introduction to the interviewers, and their biases, was not prioritised due time limitations. The participants were not informed about the individual interviewers' assumptions and personal reasons and interests in the topic. |
| **Domain 2: study design** | | | |
| ***Theoretical framework*** | | | |
| **9** | Methodological orientation and Theory | What methodological orientation was stated to underpin the study? *E.g. grounded theory, discourse analysis, ethnography, phenomenology, content analysis* | Our methodological orientation was a thematic content analysis and involved an inductive, bottom-up process (as described by Miles et al., 2020, P.62). |
| ***Participant selection*** | | | |
| **10** | Sampling | How were participants selected? *E.g. purposive, convenience, consecutive, snowball* | Sampling strategy in both Cases was self-selection. The researcher reached out to all eligible employees via email, calling for volunteers to participate. |
| **11** | Method of approach | How were participants approached? E*.g. face-to-face, telephone, mail, email* | The participants in both cases were approached via email. |
| **12** | Sample size | How many participants were in the study? | In total, 33 managers were interviewed (Case 1: 8; Case 2: 25). Of the 33 participants, 13 were interviewed individually, while the rest were interviewed in groups of 2-5 participants. The number of participants per interview in each case is presented below:   \| **Number of participants per interview** \| **Number of interviews: Case 1** \| **Number of interview: Case 2** \| **Number of participants** \| \| --- \| --- \| --- \| --- \| \| 1p \| 8 \| 5 \| 13 \| \| 2p \| - \| 2 \| 4 \| \| 3p \| - \| 1 \| 3 \| \| 4p \| - \| 2 \| 8 \| \| 5p \| - \| 1 \| 5 \| |
| **13** | Non-participation | How many people refused to participate or dropped out? Reasons? | All managers who had volunteered participated in Case 1. Non-participation in Case 2 included 3 participants who reported a need to prioritise other tasks. |
| ***Setting*** | | | |
| **14** | Setting of data collection | Where was the data collected? E*.g. home, clinic, workplace* | The location of the interviews varied: some were held in a meeting room at the workplace (n=17) and others via phone or video-conference (n=16). All participants were interviewed during working hours. |
| **15** | Presence of non-participants | Was anyone else present besides the participants and researchers? | A consultant supported the interviewer in facilitation in Case 2. |
| **16** | Description of sample | What are the important characteristics of the sample? *E.g. demographic data, date* | Data was collected 12-18 months post-relocation (Case 1: March 2017; Case 2: Nov 2019 - March). Demographic data is presented in the manuscript. |
| ***Data collection*** | | | |
| **17** | Interview guide | Were questions, prompts, guides provided by the authors? Was it pilot tested? | The interview questions are presented in Appendix 3. The questions were pilot tested. |
| **18** | Repeat interviews | Were repeat interviews carried out? If yes, how many? | No. |
| **19** | Audio/visual recording | Did the research use audio or visual recording to collect the data? | Audio recordings were made of all interviews |
| **20** | Field notes | Were field notes made during and/or after the interview or focus group? | No. The researchers relied mainly on transcribing the recordings. |
| **21** | Duration | What was the duration of the interviews or focus group? | Between 50 – 85 minutes in Case 1 (average 68 minutes)  Between 50 – 145 minutes in Case 2 (average 82 minutes). |
| **22** | Data saturation | Was data saturation discussed? | Reflections on data saturation are reported in methodological discussions in the manuscript. |
| **23** | Transcripts returned | Were transcripts returned to participants for comment and/or correction? | No. Returning the transcripts was deemed unfeasible due to delays between data collection and transcriptions. |
| **Domain 3: analysis and findings** | | | |
| ***Data analysis*** | | | |
| **24** | Number of data coders | How many data coders coded the data? | 3 coders |
| **25** | Description of the coding tree | Did authors provide a description of the coding tree? | Yes, the coding tree is provided in Appendix 3. |
| **26** | Derivation of themes | Were themes identified in advance or derived from the data? | All themes were derived from the data. |
| **27** | Software | What software, if applicable, was used to manage the data? | N/A. The data was coded in Word. |
| **28** | Participant checking | Did participants provide feedback on the findings? | No, mainly due to a delay between data-collection and analysis. |
| ***Reporting*** | | | |
| **29** | Quotations presented | Were participant quotations presented to illustrate the themes / findings? Was each quotation identified? E*.g. participant number* | Yes, quotations are presented to illustrate the findings with a participant number. |
| **30** | Data and findings consistent | Was there consistency between the data presented and the findings? | Yes |
| **31** | Clarity of major themes | Were major themes clearly presented in the findings? | Yes |
| **32** | Clarity of minor themes | Is there a description of diverse cases or discussion of minor themes? | The results portray the common themes between two cases, with a few exceptions where diversity between cases is described. |
