# Supplementary material for: Navigating productivity dilemmas and conflicting loyalties in activity-based flexible offices - A qualitative study of managers’ perspectives and coping strategies
Source: PLoS One. 2025 Nov 21;20(11):e0335945. doi: 10.1371/journal.pone.0335945 (PMC12637956; doi:10.1371/journal.pone.0335945)
Supplement: Appendix 2 — (DOCX) [file pone.0335945.s002.docx]

**Supporting information: Appendix 2. Questions used for the semi-structured individual and group interviews with the managers**

| **TOPIC** | **SAMPLE QUESTIONS** |
| --- | --- |
| Managers’ work conditions | - How do you like working in the AFO? - What are the pros and cons of working in AFOs as a manager? - How well does the AFO setting support your tasks as a manager? - Do you feel healthy and productive? - What spaces do you prefer to use in the AFO? - Do you work elsewhere (e.g. home) when carrying out your tasks? |
| Staff work conditions | - How well does the AFO support the tasks of your staff? - How do they like working in the AFO? - Have you perceived changes in the staff’s satisfaction, productivity, and health? |
| Communication strategies | - How do you find each other in the facilities? - How do you reach out to staff for individual and/or group communication? |
| Managers’ work environment responsibility | - How do you fulfil your work environment responsibilities in the AFOs? - What are the opportunities and difficulties in conducting systematic work environment management in the AFOs? - How do you make workplace adjustments for staff? |
| Leadership | - What is it like to be a leader in an AFO? - Have there been any changes in your leadership strategies? |
