# Supplementary material for: Navigating productivity dilemmas and conflicting loyalties in activity-based flexible offices - A qualitative study of managers’ perspectives and coping strategies
Source: PLoS One. 2025 Nov 21;20(11):e0335945. doi: 10.1371/journal.pone.0335945 (PMC12637956; doi:10.1371/journal.pone.0335945)
Supplement: Appendix 3 — (DOCX) [file pone.0335945.s003.docx]

**Supporting information: Appendix 3. Coding tree with main themes (numbered), sub-themes and the codes.**

| **SUB_THEMES** | **CODES** | |
| --- | --- | --- |
| 1. **Contextual preconditions shaping the managers’ experiences** | | |
| **Team distribution before relocation** | - Previously distributed teams were co-located. - Previously distributed teams remained distributed. - Previously co-located teams were dispersed. |  |
| **Number of staff** | - Easier to handle smaller control spans. |  |
| **Office type before relocation** | - Moving from open-plan offices was easier than moving from cell offices - Moving from assigned workstations posed more difficulties than moving from non-assigned stations. | |
| 1. **Misalignment between AFOs and managerial tasks** | | |
|  | **Experiences** | **Coping strategies** |
| **Perceived challenges in supporting staff on group level** | - A sense of insecurity and increased risk for intolerance among staff due to free seating, mixing with other units, and ambiguous boundaries. - Difficulties in locating each other, more asynchronous communication, limited work-related discussions. - Limited possibilities for socialisation within the unit and difficulties in creating team cohesion. - Limitations for creative tasks that required handling materials. - Limitations for meeting legal requirements for handling sensitive information. | - Changing the environmental conditions, such as finding other locations, negotiating unit-specific spaces, and adopting non-compliant behaviours. - Creating “control systems” to bring staff together, such as increased asynchronous communication, signalling availability and work location in calendars, formalising social activities, bringing together the staff via tasks instead of location, and more frequent staff meetings. |
| **Obstacles to supporting staff on individual level** | - Limitations for providing a distraction-free environment. - Difficult for managers and staff to locate each other. - Risk of compromising staffs’ privacy when providing feedback due to openness of spaces. | - Behavioural and environmental strategies to handle the distractions that staff confronted. - Passive and active strategies to cope with the loss of oversight and visual control - Communication strategies to avoid compromising staff’s integrity |
| **Productivity dilemmas** | - More available for both staff and other units - Easier to solve problems on the go - Disruptions due to increased availability, open-plan design, and clean-desking - Difficult to handle confidential tasks - Increased scrutiny and exposure of managers | - Adopting compliant and/or non-compliant behaviours for use of AFOs to allow for focused tasks - Boundary setting strategies to communicate availability - Expanding the work boundaries, e.g. work-from-home or working longer hours for focused tasks |
| 1. **Conflicting loyalties** | | |
|  | **Experiences** | **Coping strategies** |
| **Limitations for fulfilling work environment responsibilities** | - Difficult to monitor and gauge staff wellbeing and workload and risk of missing out on withdrawal signals in a free-seating arrangement - Down-prioritisation of physical work environment and transition of responsibility to staff for adjusting workstations - Increase in reported prevalence of concentration problems, more difficult to adjust compared to assigned workstations - Increased formalisation for handling workplace accommodations and unclear assignment of responsibility in the organisation for adjusting - Limitations for accommodating diversity, favouring extroverts - Risk of resignations and recruitment mistakes | - Being more observant in the facilities - Reduced engagement in absence of natural interactions - Encouraging behaviour change among staff and seeking help from occupational health and safety experts to address ergonomic issues - Ensuring undisturbed work for staff by focusing on individual behaviours, e.g. “touring with staff to help find quieter spaces” or promoting social agreements for quiet zones, seeking support from occupational health and safety experts, and formalising processes for workplace adjustments |
| **Challenges in simultaneously representing the employer and employees** | - Defending the AFO solution and the organisations’ decision despite its shortcomings - Limited information and lack of feedback from the organisation - Challenges being a spokesperson for staff and handling their frustrations when decisions were made with consideration for other units or on higher organisational levels - Being a role model and handling non-compliance with the intended use of the AFOs - Assuming ownership of the social environment despite different local interpretations of the intended use | - Being a role model for the changes - Helping staff to see positive aspects of AFOs instead of fixating on negative aspects - Compromising unit needs to ensure fairness in distribution of assigned workspaces in the organisation - Seeking formal and informal support in forums with other managers to discuss work environment problems and solutions |
